# Supplementary material for: Patterns of Intron Gain and Loss in Fungi
Source: PLoS Biol. 2004 Nov 30;2(12):e422. doi: 10.1371/journal.pbio.0020422 (PMC532390; doi:10.1371/journal.pbio.0020422)
Supplement: Table S1 — Also available at http://genes.mit.edu/NielsenEtAl/. (4.3 MB ZIP). [file pbio.0020422.st001.zip › NielsenEtAl/html/638.html]

AN5530.1.NCU07299.1.MG05375.1.FG01187.1


```
 CLUSTAL W (1.82) Multiple Sequence Alignments - Introns Inserted


Sequence 1: NCU07299.1	247 aa
Sequence 2: MG05375.1	247 aa
Sequence 3: FG01187.1	240 aa
Sequence 4: AN5530.1	237 aa
Alignment Length: 251 aa
Number Identitical Residues: 156 aa
Alignment Score (without introns) 7345


MG05375.1 	MDYQ~NRAGSKFGGGGVASHSATNADRRERLRKLALEHIDLDKDPYIFKNHLGAFECRLC
NCU07299.1	MDYQ~NRAGSKFGGGGVASHSATNADRRERLRKLALETIDLDKDPYIFRNHLGSFECRLC
FG01187.1 	MDFQ~NRAGSKFGGGGVASQSATNADRRERLRKLALETIDLDKDPYFFKNHVGSFECRLC
AN5530.1  	MDYQ0NRAGSKFGGGGVASHSATNADRRERLRKLALETIDLDKDPYFFKNHVGSFECRLC
          	**:* **************:***************** ********:*:**:*:******

MG05375.1 	LTVHQNDGSYLAHTQGRKHQTNLARRAAQEKRDGRDKNIDPQTGLPVGVVGAGFSALGLG
NCU07299.1	LTVHQNDGSYLAHTQGKKHQTNLARRAAREQKEGKGE-VDPQTGLPVGVVGAGFAALGLG
FG01187.1 	LTVHQNDGSYLAHTQGKKHQTNLARRAAREQKEGRQGAIDPATGLPIGVTGAGFAQ----
AN5530.1  	LTVHQNDGSYLAHTQGRKHQTNLARRAAREQREGKNA--DPAS-LP-GAMGV--------
          	****************:***********:*:::*:    ** : ** *. *.        

MG05375.1 	-GGARKNAVKIGRPGYKITKIRDPVTRAEGLLFQLQFPDISPGTVPKWQVMSAFSQRVEE
NCU07299.1	AGGVRKNVVKIGRPGYKITKVRDPITRQQGLLFQLQYPDIAPGVTPKWQVMSAFSQRVEE
FG01187.1 	----RRNIVKIGRPGYKITKIRDPVSRQQGLLFQLQYPDATPETSPKWQVMNAFTQHIEE
AN5530.1  	--QVRKQTIKIGRPGYKITKIRDPLTRQFGLLFQLQYQEITPGVKPRVRFMSAFEQKVEE
          	    *:: :***********:***::*  *******: : :* . *: :.*.** *::**

MG05375.1 	P-DRNYQYLLVAAEPYETCGFKVPARELDKREGRMFEYWDPDAKEYWVQVMFMTEREERF
NCU07299.1	P-DRNYQYLLVAAEPYETCGFKIPARELDKREDKQFEFWDPDSKEYWVQIMFMTEREERF
FG01187.1 	P-DKNFQYLLVAAEPYETVGFKIPARELDKREDKQFAFWDPDAKEYWIQVMFMTEREERF
AN5530.1  	PPDKNFQYLVIAAEPYQTCGFKLQAREIDRREGRYWTWFDEDSKEFWVQIMFKTEREERF
          	*.*:*:***::*****:* ***: ***:*:**.: : ::* *:**:*:*:** *******

MG05375.1 	NAAPGLGARS--
NCU07299.1	NAAPGLTGRR--
FG01187.1 	NAAPGQAMRR--
AN5530.1  	SGVPGLAPMGAA
          	...**     ::
```
